# What is the role of cardiorespiratory fitness and sedentary behavior in relationship between the genetic predisposition to obesity and cardiometabolic risk score?

**DOI:** 10.1186/s12872-022-02537-5

**Published:** 2022-03-09

**Authors:** Ana Paula Sehn, Caroline Brand, João Francisco de Castro Silveira, Lars Bo Andersen, Anelise Reis Gaya, Pâmela Ferreira Todendi, Andréia Rosane de Moura Valim, Cézane Priscila Reuter

**Affiliations:** 1 Graduate Program in Health Promotion, University of Santa Cruz do Sul (UNISC), Independência Av, 2293 – Universitário, Santa Cruz Do Sul, RS 96815-900 Brazil; 2 Faculty of Education, Arts and Sport, Westerm Norway University of Applied Sciences, Songdal, Norway; 3 Department of Sports Medicine, Norwegian School of Sport Sciences, Oslo, Norway; 4 School of Physical Education, Physiotherapy and Dance. Graduate Program in Human Movement Sciences, Federal University of Rio Grande Do Sul (UFRGS), Porto Alegre, RS Brazil; 5 Graduate Program in Endocrinology, Federal University of Rio Grande Do Sul (UFRGS), Porto Alegre, Brazil; 6 Life Sciences Department, University of Santa Cruz Do Sul (UNISC), Santa Cruz Do Sul, RS Brazil; 7 Health Sciences Department, University of Santa Cruz Do Sul (UNISC), Santa Cruz Do Sul, RS Brazil

**Keywords:** Screen time, Cardiorespiratory fitness, *FTO* polymorphism, Metabolic syndrome, Childhood, Young

## Abstract

**Background:**

Genetic factors along with inadequate lifestyle habits are associated with the development of cardiometabolic alterations. Thus, the present study aimed to examine the role of sedentary behavior on the relationship between rs9939609 polymorphism (fat mass and obesity-associated gene-FTO) and cardiometabolic risk score according to cardiorespiratory fitness (CRF) levels in children and adolescents.

**Methods:**

A cross-sectional study with 1215 children and adolescents (692 girls), aged between 6 and 17 years. Screen time as a marker of sedentary behavior was evaluated through a self-reported questionnaire and CRF was estimated using the 6-min walking and running test. The genotyping of the *FTO* rs9939609 polymorphism was performed using a real-time polymerase chain reaction. Clustered cardiometabolic risk score (cMetS) was calculated by summing z-scores of total cholesterol/high-density lipoprotein cholesterol ratio, triglycerides, glucose, systolic blood pressure, and waist circumference, and dividing it by five. Moderation analyses were tested using multiple linear regression models**.**

**Results:**

The coefficient of the interaction term of *FTO* (rs9939609) and screen time indicated that screen time was a significant moderator on the relationship between *FTO* rs9939609 polymorphism and cMetS (*p* = 0.047) in children and adolescents classified with low CRF (β = 0.001; 95% CI = 0.001; 0.002). It was observed a significant association between genotype risk (AA) of *FTO* polymorphism and cMetS, in participants that spent more than 378 min a day in front of screen-based devices (β = 0.203; 95% CI = 0.000; 0.405). No interaction term was found for those with high CRF.

**Conclusions:**

High sedentary behavior seems to influence the relationship between genetic predisposition to obesity and cardiometabolic risk factors in children and adolescents with low CRF.

## Background

Cardiometabolic risk factors are already observed at early ages mainly by the presence of obesity, lipid profile alteration, and high blood pressure levels, causing a worrying scenario at the public health level [1–3]. Environmental, behavioral, and genetic factors, along with the essential role of lifestyle habits, such as sedentary behavior, sleep duration, eating habits, and physical activity are associated with these cardiometabolic alterations [4, 5].

In children and adolescents, the genetic predisposition is one of the aspects related to obesity [6] and the development of metabolic complications [7, 8] and in adults is linked to the development of hypertension, dyslipidemia, and type 2 diabetes [6, 9]. Among obesity-associated genetic factors, the rs9939609 single nucleotide polymorphism (SNP), located on fat mass and obesity-associated gene (*FTO*), has been widely investigated in youth and established as exerting a direct influence on adiposity indicators [10–12]. Given the knowledge that adiposity plays a central role in the origin of cardiometabolic disease [13], it is important to understand the relationship between fat mass, rs9939609 (*FTO*) polymorphism and cardiometabolic risk factors at early ages.

Apart from the genetic contribution, lifestyle habits, such as sedentary behavior, have been associated with obesity and cardiometabolic complications, in which more hours in front of screen-based devices increase the cardiometabolic risk score [14–16]. Recently, the World Health Organization recommended that children and adolescents should reduce time spent being sedentary. Additionally, the same guideline suggests that children and adolescents should accomplish at least an average of 60 min per day of moderate-to-vigorous physical activity [17–19], which exerts a direct effect on physical fitness [20], an important health indicator during childhood and adolescence [21]. Cardiorespiratory fitness (CRF) is an important component of physical fitness, and its positive effects on cardiometabolic health has been widely described in the literature [22–25].

Therefore, given the knowledge that the genetic predisposition to obesity is associated with sedentary behavior, as well as CRF [12, 26–28], we intend to investigate the cardiometabolic health by understanding the inter-relationships between predisposition to obesity and sedentary behavior. Also, we hypothesized that CRF could exert a protective role in this context, once there is evidence supporting that better fitness levels seem to attenuate deleterious outcomes to cardiometabolic health associated with higher adiposity [29], and even attenuate the potential development of excess weight amongst those with a genetic predisposition [12, 26]. Thus, the present study aimed to examine the role of sedentary behavior on the relationship between *FTO* rs9939609 polymorphism and cardiometabolic risk score according to CRF levels in children and adolescents.

## Methods

This cross-sectional study includes data from a cohort study developed in public and private schools in a city in Southern Brazil, carried out in the following phases and years: Phase I (2004–2005), Phase II (2007–2009), Phase III (2011–2012), Phase IV (2014–2015), and Phase V (2016–2017). In 2004, twenty-five schools were randomly selected from a total of fifty schools with 20,380 schoolchildren. Students of all regions of the city were considered to calculate the population density of students to be included in the research. All students from the 25 schools were invited to participate. Phase IV data was used in the present cross-sectional study and included 1215 children and adolescents, aged between 6 and 17 years. The sample size calculation for the present study was done through the software G*Power version 3.1. A small effect size (f^2^ = 0.03), statistical power of 0.80, alpha (type I error rate) of 0.05, and 6 predictors were considered. The minimum number of participants was established as 461. However, to avoid probable difficulties with sample loss, an increase of 10% was assumed, totaling 507 for each group (low and high CRF) [30]. For the present study we included only individuals with full data on blood collection and CRF, whereas individuals that did not present information about screen time and blood pressure were excluded. This study was approved by the University of Santa Cruz do Sul research ethics committee (nº 1.498.305), and it was conducted following the Resolution 466/2012 of the National Council of Health in Brazil. The schoolchildren’s parents or legal guardians signed free and informed consent forms.

All variables included in the present study were assessed at the University of Santa Cruz do Sul by a trained research team. A self-reported questionnaire was used to determine lifestyle habits and skin color. The parents of the children aged between 6 to 10 years helped them to fulfill the questionnaire. Screen time as a marker of sedentary behavior was evaluated with the following question: “How much time in minutes do you spend in front of the TV, computer, and videogame per day?”. The total time spent on all three screen-based devices was summed. Sleep duration was determined according to the questions: “What time do you go to sleep during the week and the weekend?”; and “What time do you get up during week and weekend?”. The total hours of sleeping per week was calculated, and a mean of sleep duration per day was established. To determine skin color participants should indicate one of the following options: white, black, brown/mulatto, indigenous, or yellow.

Body weight and height were evaluated. An anthropometric scale with a coupled stadiometer (Filizola®, the precision of 0.01 kg and 0.01 cm, respectively) was used. The waist circumference (WC) was measured using an inelastic tape with a resolution of 1 mm (Cardiomed®) placed on the narrowest part of the trunk between the last rib and the iliac crest. Tanner´s criteria was adopted to evaluate sexual maturation [31]. The researcher explained the different stages of the pictures and the participant indicated the correspondent picture accordingly to their current stage, considering breast development for girls, genital development for boys, and pubic hair for both. Then, five stages of sexual maturation were determined and subsequently categorized into four classes: prepubertal (stage I), initial development (stage II), continuous maturation (stages III and IV), and matured (stage V).

The estimate of CRF followed the 6-min walking and running test procedures established by the *Projeto Esporte Brasil* [32]. The test was performed on an athletic track and consisted of participants instructed to run as long as possible within the established time to accomplish the greatest number of turns. The total distance in meters was calculated. Subsequent quantification of the total distance covered by the participants in meters was used to classify CRF into low or high levels (risk and healthy, respectively), according to sex- and age-cutoff points [32], in which it was considered as low levels results below of cutoff points and high levels values above of cutoff points.

Total cholesterol (TC), high-density lipoprotein cholesterol (HDL-C), triglycerides (TG), and glucose were evaluated via blood samples, which were collected early in the morning after a 12 h-fasting, using serum samples and commercial kits (DiaSys Diagnostic Systems, Germany), performed on Miura 200 automated equipment (I.S.E., Rome, Italy). For intern quality control was used normal control (Topkon N, Kovalent) and altered (Topkon P Kovalent). The analytes were dosed respecting the following intra and inter assay variation coefficients: TC (1.30% and 2.4%), HDL-C (0.75% and 1.80%), TG (1.8% and 2.20%) and glucose (0.92% and 2.5%). Systolic blood pressure (SBP) was assessed through the auscultatory method, using a sphygmomanometer and a stethoscope following the protocols of the VI Guidelines of the Brazilian Society of Cardiology [33]. Two measures were performed on the left arm using a cuff appropriated to the participant arm circumference, after five minutes of resting. The lowest result for SBP was considered.

The cardiometabolic risk was assessed using a clustered cardiometabolic risk score (cMetS), which was calculated by summing z-scores of TC/HDL-C ratio, TG, glucose, SBP, and WC, and dividing it by five. Sex- and age-specific standardized z-scores were calculated using international references for each risk factor with the following equation: z-score = ([X—${\overline{\text{X}}}$]/SD); where X is the continuous value observed for the risk factor; ${\overline{\text{X}}}$ is the predicted mean calculated for the risk factor using regression equations, and SD is the standard deviation of the international reference [34]. Before analysis, skewed variables (TC/HDL-C ratio, TG, and WC) were transformed by the natural logarithm.

To evaluate *FTO* rs9939609 polymorphism, DNA samples were extracted from whole blood stores with EDTA using the Qiagen Kit (QIAamp DNA Blood Mini Kit, Qiagen™, Germany). DNA was quantified using a Qubit^VR^ 2.0 Fluorometer (Invitrogen, Carlsbad, CA). The genotyping of the polymorphism was performed using real-time polymerase chain reaction (PCR) allelic discrimination assays in a 96-well plate; the reactions were performed in 10 ml volume, with 10 ng of genomic DNA as a template. TaqMan™ assay C_30090620_10 (RS9939609) and Master Mix PCR Universal were purchased from Applied Biosystems (Foster City, CA, USA). The genotyping was performed in duplicate, showing an accuracy of 100%. The choice of the evaluated polymorphism was the result of a literature search and an evaluation of allelic frequency using HapMap (The International HapMap Consortium,2007).

### Statistical analysis

Descriptive statistics is presented by means and standard deviations. The bootstrapping resampling procedure for the independent two-tailed *t* test was used to examine differences of continuous variables and the chi-squared test was used to examine difference for categorical variables. The bootstrapping procedure used a resampling of 1000 bootstrap samples and the Bias Corrected Accelerated (BCa) method. Standardized differences (Cohen’s *d*) were also calculated for continuous variables. The values of *d* < 0.39 indicated a small difference; 0.40 < *d* < 0.79 indicated a medium difference; and *d* > 0.80 indicated a large difference [35].

Moderation analyses were tested through multiple linear regression models using the PROCESS macro for the Statistical Package for Social Sciences (SPSS) version 23.0 (IBM Corp). The moderation analysis tested the following relationships: the direct association of the predictor (*FTO*—rs9939609) on cMetS; the direct association of the moderator (screen time) on cMetS; and the interaction association between predictor (*FTO*—rs9939609) and moderator (screen time) on cMetS. These models were applied according to high and low CRF levels. In order to further characterize the moderation role that screen time presented on the relationship between *FTO* rs9939609 polymorphism and cMetS, we applied the Johnson–Neymann technique. This technique allows the determination of associations between the independent variable *(FTO* rs9939609) and dependent variable (cMetS) across all levels of the moderator variable (low, middle, and high screen time, given by 16^th^, 50^th^, and 84^th^ percentiles, respectively) [36]. The moderation models were adjusted for skin color, sexual maturation, and sleep duration. The AT + TT genotype was the reference category for these analyses. Effect sizes (f^2^) were calculated for the multiple linear regression models using the following equation: f^2^ = r^2^_adjusted_ / (1 – r^2^_adjusted_); where r^2^_adjusted_ was the proportion of variance in the dependent variable that can be explained by the independent variables in the models and it was obtained from the moderation analyses. The probability value of *p* < 0.05 was considered to be significant in all analysis.

## Results

Children’s and adolescents’ characteristics across different genotypes are presented in Table 1. Individuals with the genotype of risk (AA) demonstrated higher WC (small difference), whereas, 13.1% of the whole sample presented AA genotype risk allele.

**Table 1:** Descriptive characteristics of children and adolescents across different genotypes

	All (n = 1215)	AT + TT genotype (n = 1057)	AA genotype (n = 158)	Standardized difference (Cohen’s *d*)
**Mean (SD)**				
Age (years)	12.36 (2.83)	12.35 (2.83)	12.43 (2.85)	0.03
Weight (kg)	48.27 (15.82)	47.89 (15.31)	50.79 (18.74)	0.18
Height (m)	1.52 (0.15)	1.52 (0.15)	1.53 (0.16)	0.07
Screen time (min/day)	242.44 (177.66)	238.56 (175.41)	268.38 (190.58)	0.17
Sleep duration (min/day)	122.35 (195.14)	558.05 (96.18)	555.88 (96.76)	0.02
Triglycerides (mg/dL)	73.83 (37.68)	73.23 (35.97)	77.83 (47.52)	0.12
Total cholesterol (mg/dL)	158.85 (33.12)	158.86 (32.71)	158.80 (35.86)	< 0.01
High-density lipoprotein cholesterol (mg/dL)	62.59 (11.37)	62.69 (11.34)	61.97 (11.58)	0.06
Glucose (mg/dL)	89.34 (12.04)	89.37 (12.34)	89.16 (9.79)	0.02
TC/HDL-C	2.59 (0.62)	2.59 (0.60)	2.63 (0.74)	0.07
Systolic blood pressure (mmHg)	106.24 (14.98)	105.95 (14.91)	108.13 (15.39)	0.15
Waist circumference (cm)	67.30 (10.60)	67.00 (10.19)	69.29 (12.88)	0.22*
cMetS	−0.09 (0.74)	−0.10 (0.74)	−0.03 (0.74)	–
**n (%)**				
**Sex**				
Male	523 (43.0)	448 (42.4)	75 (47.5)	–
Female	692 (57.0)	609 (57.6)	83 (52.2)	
**CRF**				
Healthy	526 (43.3)	449 (42.5)	77 (48.7)	–
Risk	689 (56.7)	608 (57.5)	81 (48.7)	
**Skin color**				
White	921 (75.8)	802 (75.9)	119 (75.3)	–
Black	96 (7.9)	87 (8.2)	9 (5.7)	
Mixed race-Brown/mulatto	178 (14.7)	150 (14.2)	28 (17.7)	
Others races (indigenous/yellow)	20 (1.6)	18 (1.7)	2 (1.3)	
**Maturational stage**				
Pre-pubertal	260 (21.4)	233 (22.0)	27 (17.1)	–
Initial development	299 (24.6)	254 (24.0)	45 (28.5)	
Continuous maturation (stage III and IV)	549 (45.2)	479 (45.3)	70 (44.4)	
Maturated	107 (8.8)	91 (8.6)	16 (10.1)	

*SD* standard deviation, *n* absolute frequency, *%* relative frequency, *CRF* cardiorespiratory fitness, *cMets* clustered cardiometabolic risk score (sum of total cholesterol/high-density lipoprotein cholesterol ratio, triglycerides, glucose, systolic blood pressure, and waist circumference z-scores, divided by five), *FTO* fat mass and obesity-associated polymorphism

*Statistically difference between genotypes using the bootstrapping resampling procedure for the independent two-tailed *t* test (*p* < 0.05). ** Statistically difference between genotypes using the Chi-square test (*p* < 0.05)

^a^Significance level, according to the chi-squared test for evaluation of Hardy–Weinberg equilibrium (*p* = 0.826) when comparing the expected values (163—AA; 564—AT; and 488—TT) and observed values (158—AA; 574—AT; and 483—TT)

Table 2 presents the moderator role of screen time in the relationship between rs9939609 *FTO* polymorphism and cMetS in children and adolescents across CRF levels. The coefficient of the interaction term of *FTO* (rs9939609) and screen time indicated that screen time was a significant moderator on the relationship between *FTO* rs9939609 polymorphism and cMetS (*p* = 0.047) in children and adolescents classified as low CRF.

**Table 2:** Screen time, rs9939609 FTO polymorphism and cMetS in children and adolescents with different CRF levels

	cMetS	
	β (95% CI)	*p*
Screen time	−0.001 (−0.002; 0.001)	0.059
**Low CRF**		
*FTO* (rs9939609)		
AT + TT genotype	1	
AA genotype (risk allele)	−0.151 (−0.453; 0.152)	0.328
Screen time × *FTO* (rs9939609)	0.001 (0.001; 0.002)	0.047
Effect size (f^2^)	0.01	
**High CRF**		
Screen time	0.001 (−0.001; 0.002)	0.184
*FTO* (rs9939609)		
AT + TT genotype	1	
AA genotype (risk allele)	0.227 (−0.041; 0.495)	0.096
Screen time × *FTO* (rs9939609)	−0.001 (−0.001; 0.001)	0.162
Effect size (f^2^)	0.03	

*CI* confidence interval, *FTO* fat mass and obesity-associated polymorphism, *CRF* cardiorespiratory fitness, *cMetS* clustered cardiometabolic risk score (sum of total cholesterol/high-density lipoprotein cholesterol ratio, triglycerides, glucose, systolic blood pressure, and waist circumference z-scores, divided by five); All models were adjusted for sexual maturation, skin color, and sleep duration

**Fig. 1:**
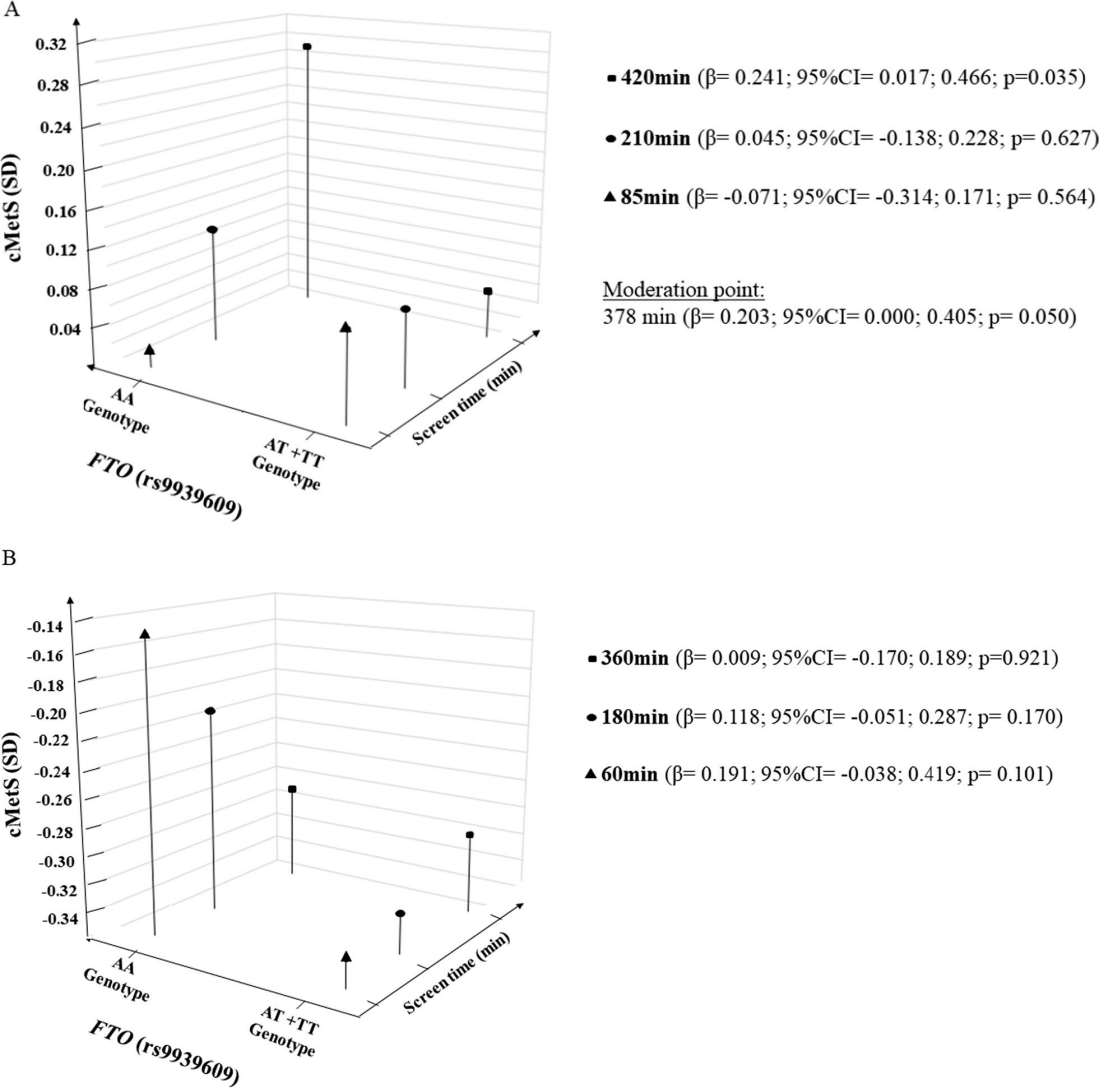
Role of screen time on the relationship between rs9939609 *FTO* polymorphism and cMetS. **A** Children and adolescents with low CRF; **B** children and adolescents with high CRF; FTO: fat mass and obesity-associated rs9939609 polymorphism; cMetS: clustered cardiometabolic risk score (sum of total cholesterol/high-density lipoprotein cholesterol ratio, triglycerides, glucose, systolic blood pressure, and waist circumference z-scores, divided by five); β: linear regression coefficient; CI: confidence interval; SD: standard deviation. The category AT + AA was considered the reference group for all analyses. Model adjusted for skin color, sexual maturation, and sleep duration

The Johnson–Neymann technique was applied for the low CRF model that presented a significant interaction, indicating that the association between *FTO* (rs9939609) and cMetS varied according to low, middle, and high screen time levels (Fig. 1A). It was observed a significant association between genotype risk (AA) of *FTO* polymorphism and cMetS for participants that spent more than 378 min a day in front of screen-based devices. Additionally, Fig. 1B presents the model (high CRF) wherein a significant interaction was not found.

## Discussion

The main findings of the present study indicate that screen time exhibited a moderator role in the relationship between *FTO* rs9939609 polymorphism and cMetS only in children and adolescents with low CRF. Therefore, it seems that high CRF possibly attenuates the association between FTO rs9939609 polymorphism, sedentary behavior, and cMetS. Further, the association between genotype risk (AA) of *FTO* polymorphism and cMetS of those with low CRF levels was dependent on high time spent in front of screen-based devices (378 min). Hence, it seems that the association between genetic predisposition to obesity and cMetS is evident only in individuals with high sedentary behavior and low CRF levels.

Studies have stated that higher (less favorable) adiposity levels during childhood and adolescence are a great risk factor for an unhealthy cardiometabolic status [25, 37]. Further, a previous systematic review demonstrated that prolonged sedentary behavior is associated with deleterious risk factors, leading to worse cardiometabolic health during childhood and adolescence [38], even for individuals with a genetic predisposition to obesity [27, 28]. It is plausible that the mechanisms linking sedentary behaviors and having a predisposition to obesity can lead to worse cardiometabolic health, but the findings from the present study seem to suggest that it only happens amongst those with a worse CRF.

With respect to the role of sedentary behaviors and physical activity mechanisms, there is evidence suggesting that sedentarism is not independently associated with cardiometabolic health regardless of physical activity levels, especially moderate-to-vigorous physical activity [39], which could partially explain why those participants with high CRF in the present sample seemed to be ‘protected’ against the development of less favorable cardiometabolic risk factors. Addittionally, there is evidence linking time being sedentary and worse cardiometabolic health via poor dietary patterns. Evidence suggests that snacking during sedentary time may be as important as sedentarism itself [40]. However, snacking was not considered within our models.

A systematic review and meta-analysis evaluating the prospective associations between sedentary time in youth and cardiometabolic health later in life did not find any evidence supporting this association [41]. On the other hand, in agreement with the aforementioned statements and findings, it seems that as long as children and adolescents spend great amounts of time in moderate-to-vigorous physical activity, being sedentary causes little harm to cardiometabolic overall health [41]. To our knowledge, there are few studies evidencing the influence of screen time and CRF on the relationship between genetic predisposition to obesity and cardiometabolic risk. The literature has only demonstrated independent associations for genetic predisposition [12, 26–28] and cardiometabolic alterations [14, 15, 22–24, 42] with high sedentary behavior time and worse CRF levels.

Thus, understanding the complex relationships between engagement in physical activities (especially those with higher CRF demand) and sedentary behaviors, and considering the predisposition to obesity is an extremely important need, once these lifestyle behaviors and adiposity parameters tend to persist from childhood and adolescence into adulthood [43–46], which can lead to chronic diseases development and higher morbidity and mortality risk. Our findings support and suggest the importance of increasing physical activity engagement, such that CRF levels are improved [47], and reducing the amounts of time spent in sedentary activities looking at cardiometabolic health. Further, interventions targeting these behaviors should start as soon as possible at an early age, especially amongst those with a genetic predisposition to obesity.

Our study has some worthwhile strengths that should be mentioned. This is the first study presenting the role of high sedentary behavior on the relationship between genetic predisposition and cardiometabolic risk considering the CRF levels of children and adolescents. In addition, we know that the evidence indicates that CRF is an important factor to be considered when referring to adiposity once better CRF values are associated with lower body weight, due to this, our findings indicated that CRF assumes an important role since seems to minimize the negative influence of genetic predisposition. Thus, in the present study increased CRF is associated with reduction of percent body fat in children and adolescents with overweight and obesity as indicated by García-Hermoso et al. [48]. We also highlight the importance of limiting time spent on sedentary behaviors, indicating an objetive recommendation on this issue, as well as practicing at least 60 min a day of physical activity for a better cardiometabolic health in childhood. However, some limitations should also be mentioned like the use of a self-reported questionnaire to evaluate sedentary behavior, which could have biased this variable. Also, the use of a submaximal exercise field test to estimate CRF levels. The cross-sectional nature of the study, not allowing to determine cause and effect. Moreover, physical activity was not included in the analysis once it was not evaluated in these individuals. Finally, the evaluation of only one genetic polymorphism; once we know that many other genes are related to obesity [49].

## Conclusion

In conclusion, high sedentary behavior seems to influence the relationship between genetic predisposition to obesity and cMetS in children and adolescents with low CRF. In addition, appropriate CRF levels can attenuate the influence of high screen time and genetic predisposition on cMets in pediatric population. Therefore, physical fitness is an important indicator of cardiometabolic health, once seem to minimize the deleterious effects of sedentary behavior in this population. Thus, actions that promote active behaviors and identify individuals with a genetic predisposition to obesity at an early age are important to prevent cardiometabolic disease.

## Data Availability

The database used and analyzed in the present study is not publicly available as its information may compromise the participants' privacy and consent involved in the research. However, the data are available from the corresponding author, upon request.

## Abbreviations

BCa: Bias corrected accelerated
cMetS: Clustered cardiometabolic risk score
CRF: Cardiorespiratory fitness
FTO: Fat mass and obesity-associated gene
HDL-C: High-density lipoprotein cholesterol
SBP: Systolic blood pressure
SNP: Single nucleotide polymorphism
SPSS: Statistical package for social sciences
TC: Total cholesterol
TG: Triglycerides
WC: Waist circumference
